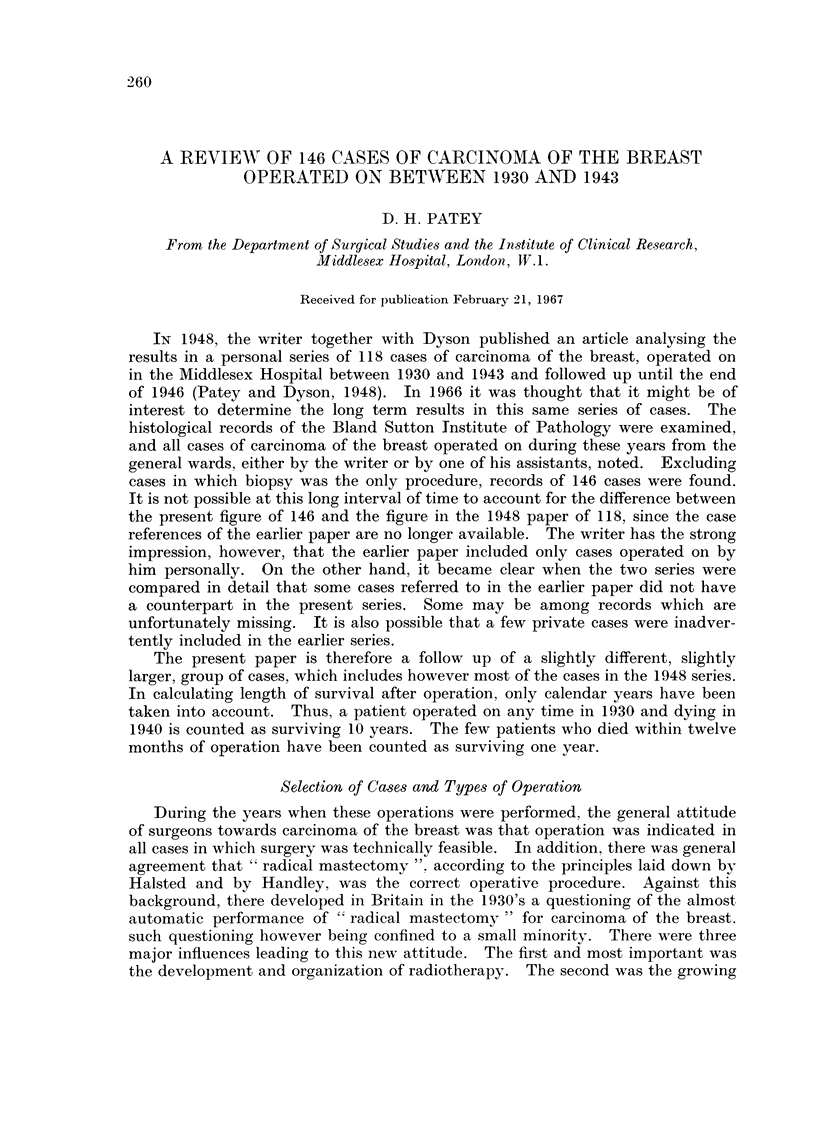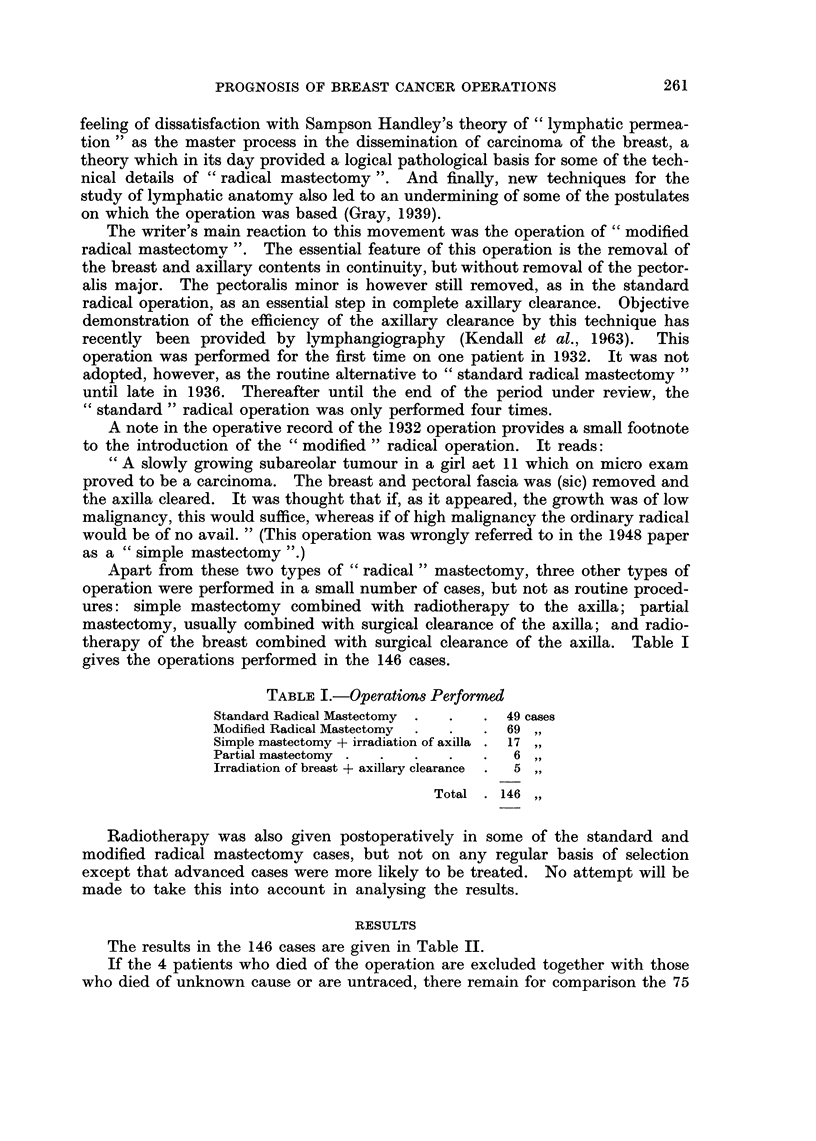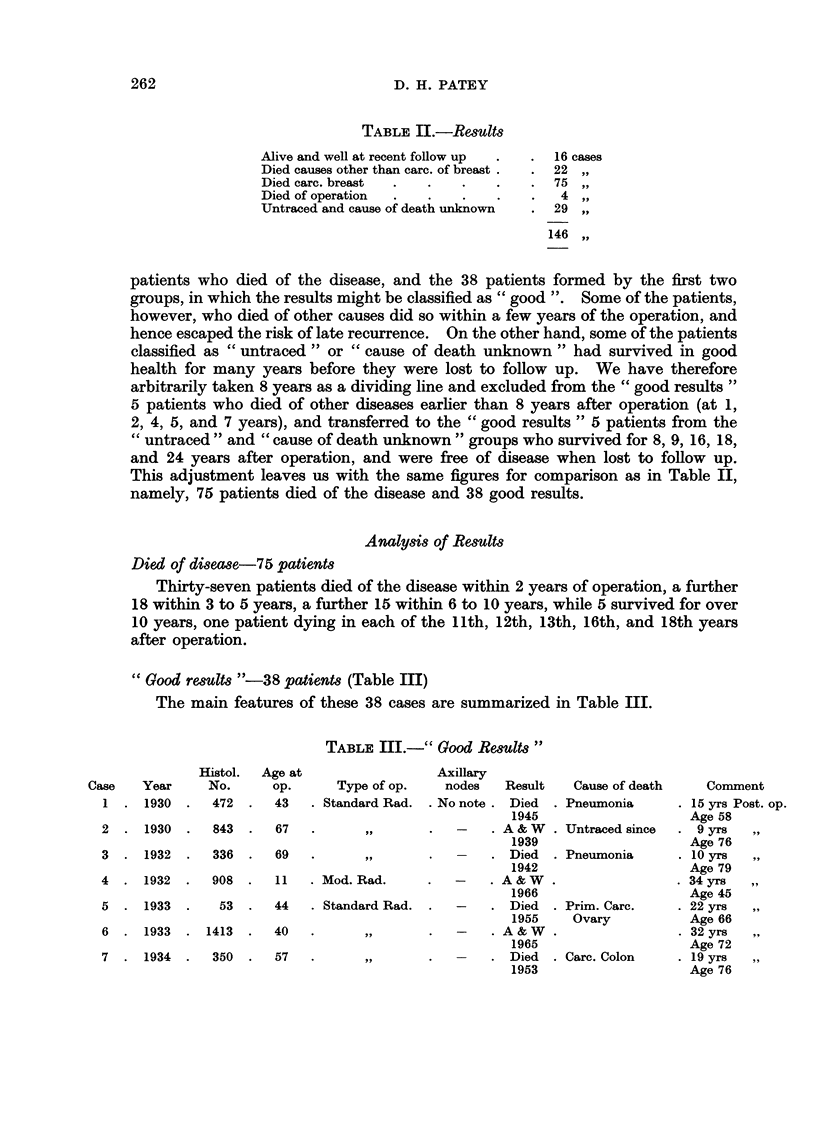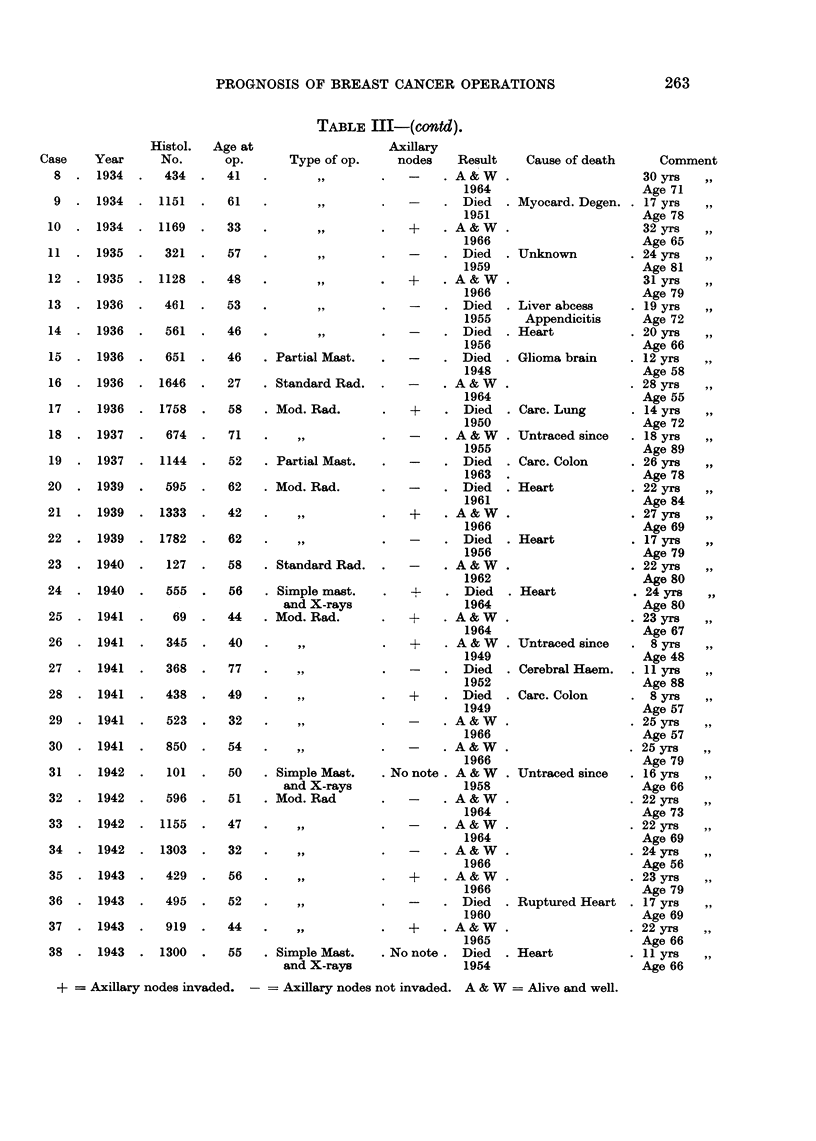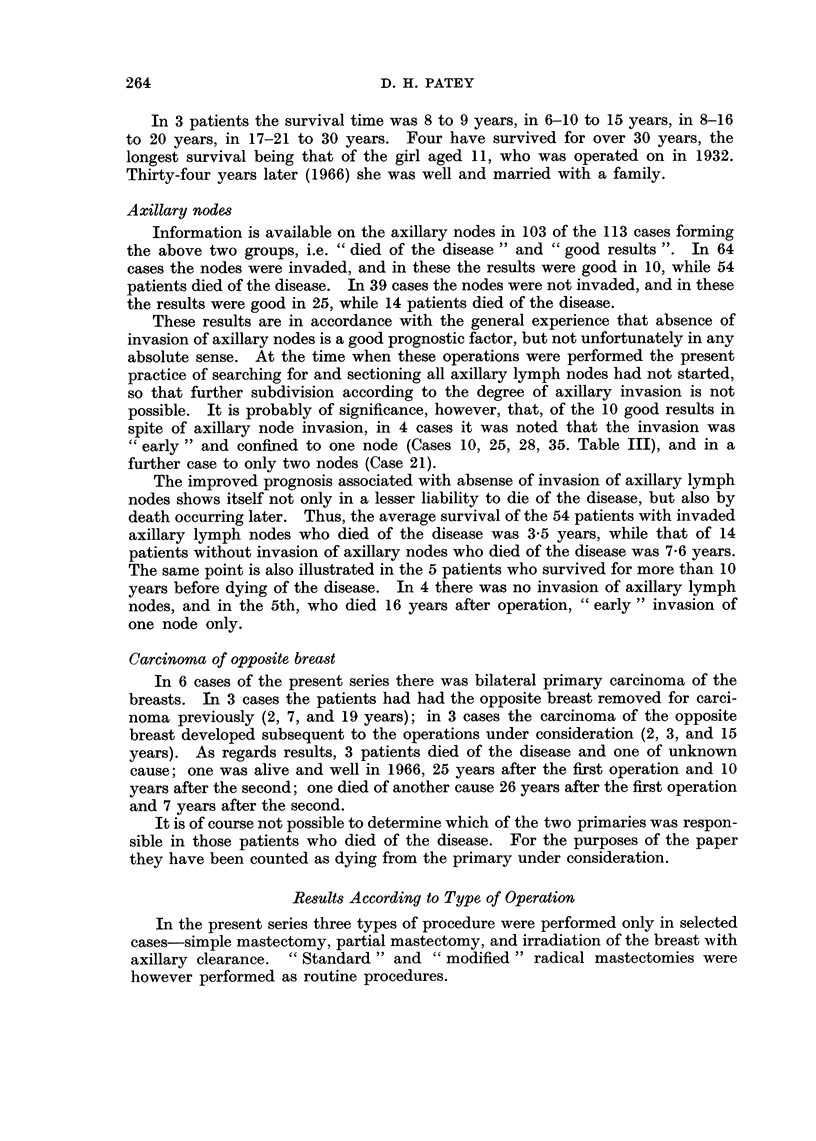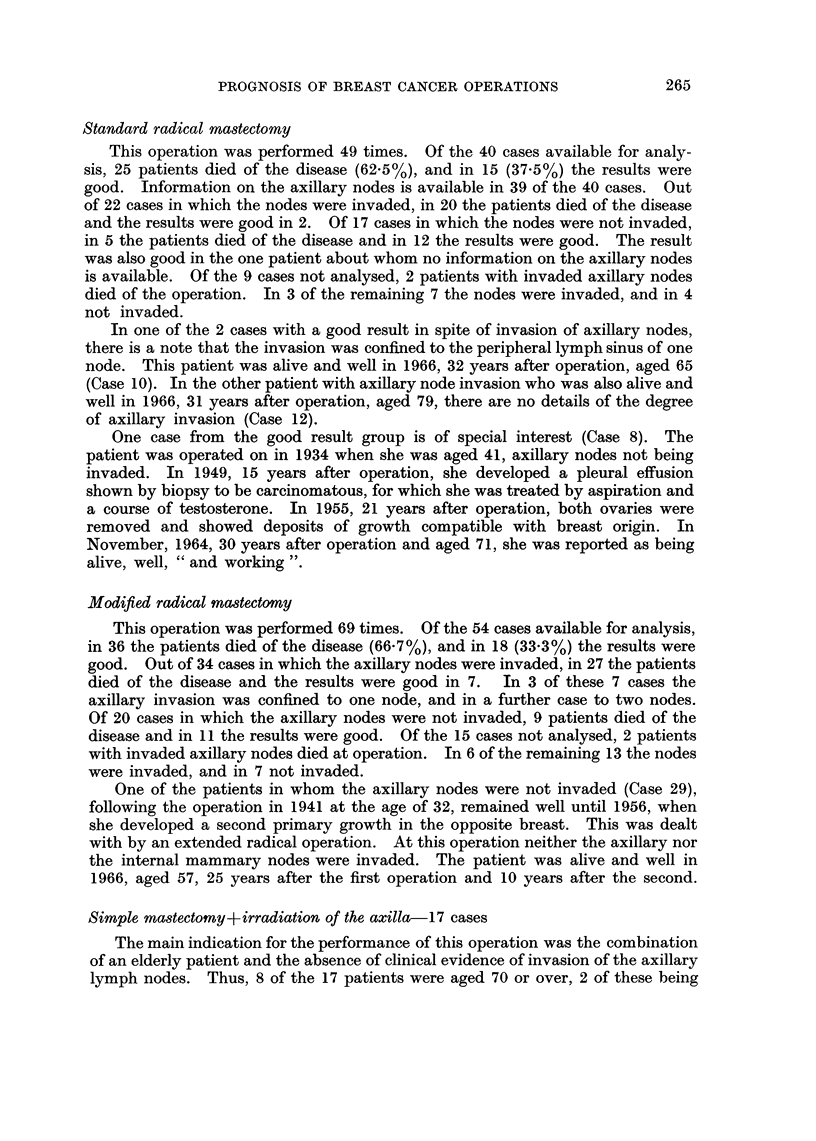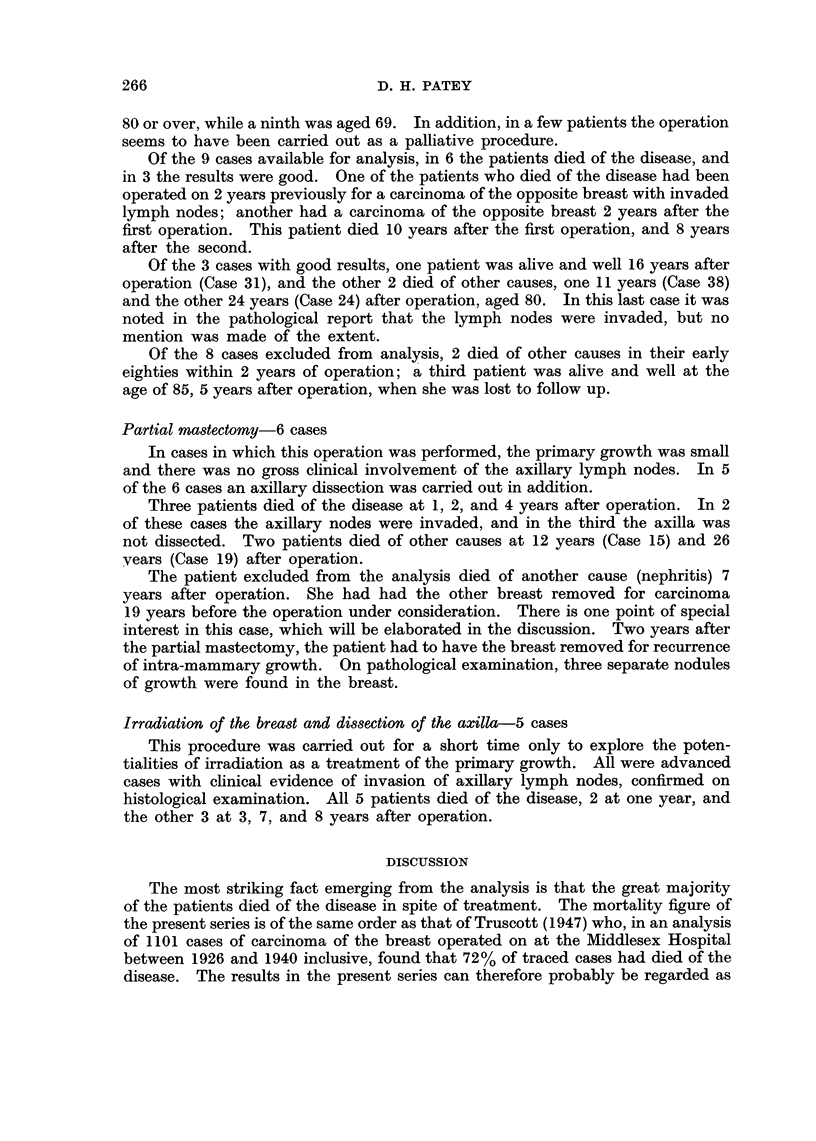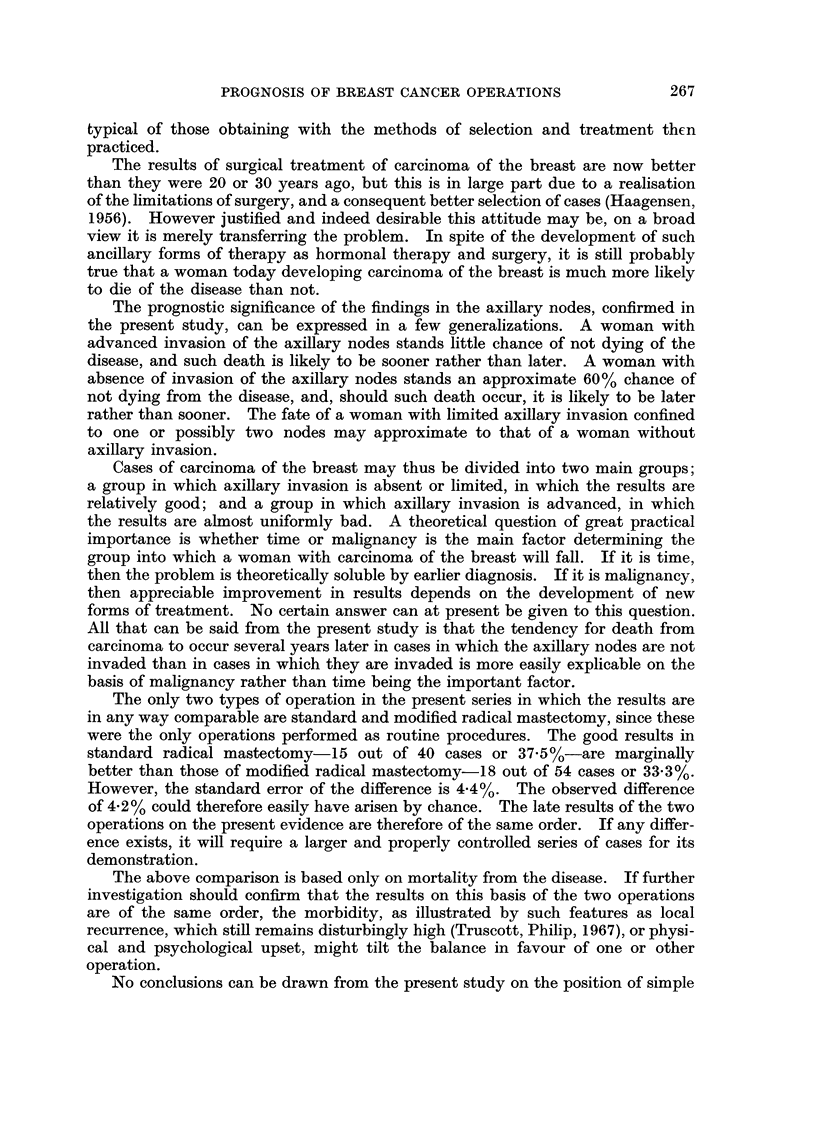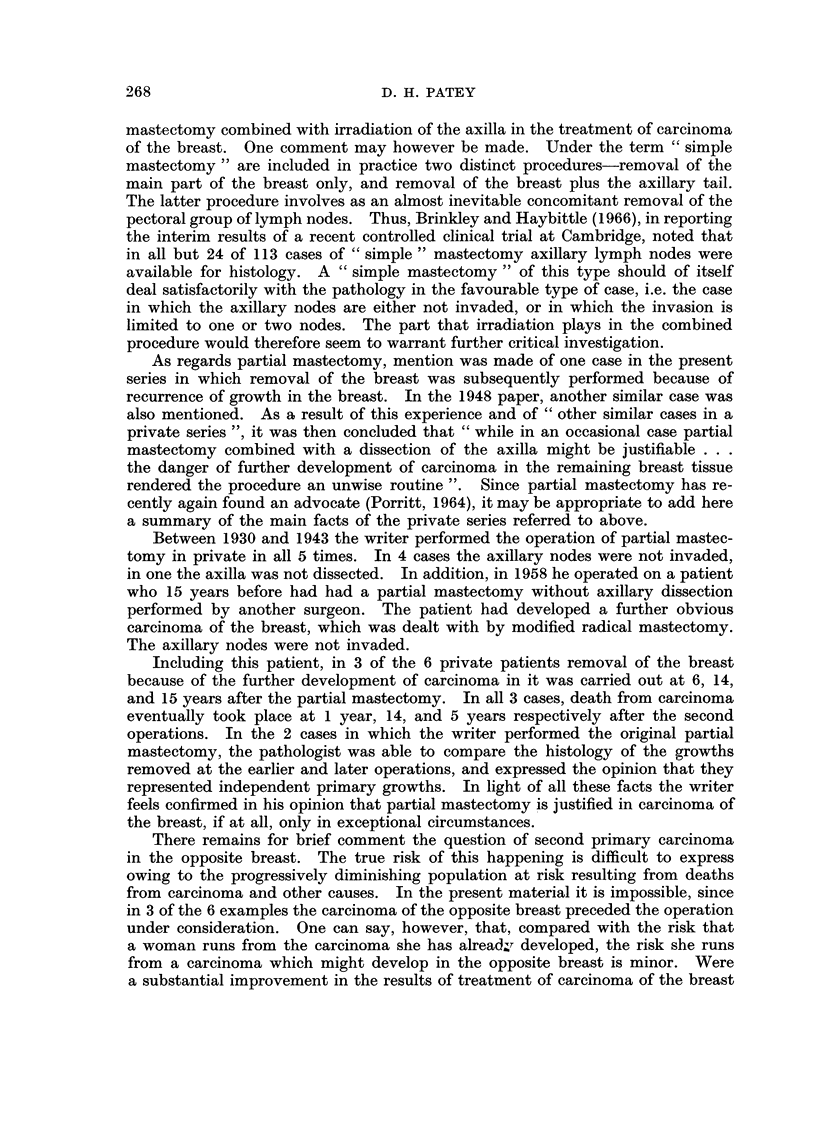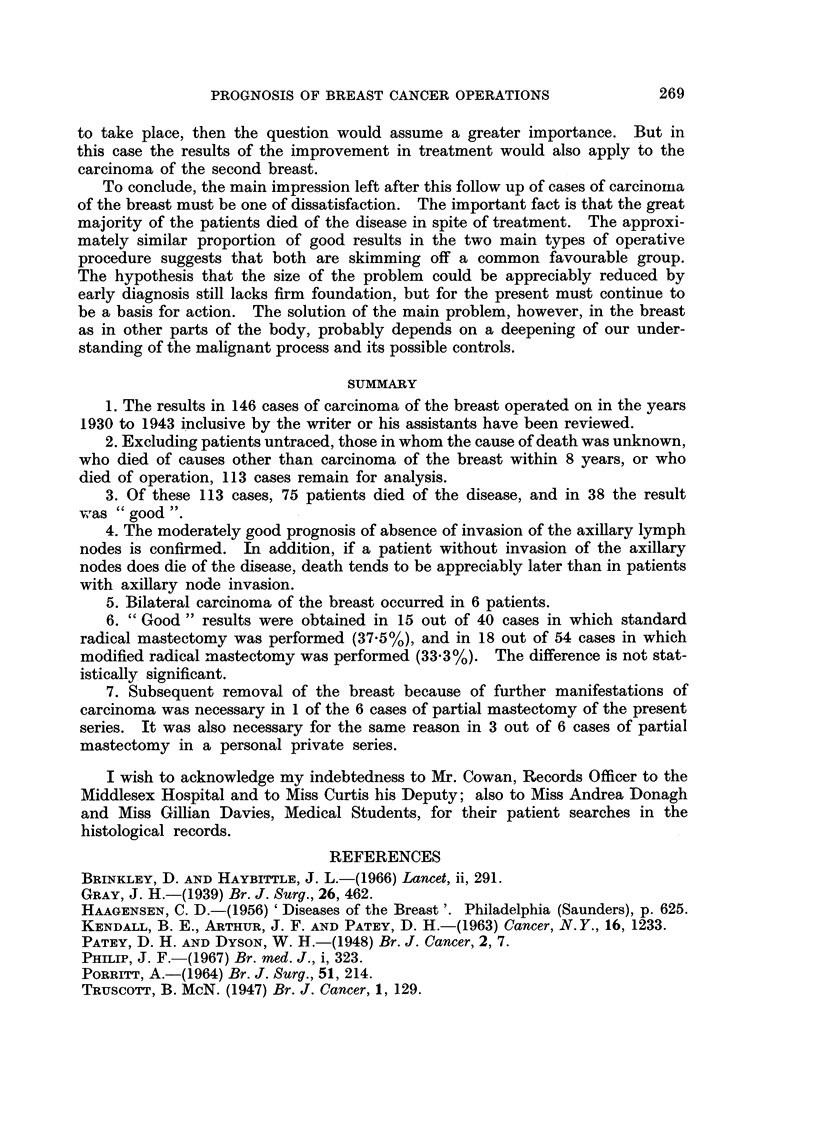# A review of 146 cases of carcinoma of the breast operated on between 1930 and 1943.

**DOI:** 10.1038/bjc.1967.28

**Published:** 1967-06

**Authors:** D. H. Patey


### A REVIEW OF 146 CASES OF CARCINOMA OF THE BREAST OPERATED ON BETWEEN 1930 AND 1943

D. H. PATEY

*From the Department of Surgical Studies and the Institute of Clinical Research, Middlesex Hospital, London, W.1.*

Received for publication February 21, 1967

In 1948, the writer together with Dyson published an article analysing the results in a personal series of 118 cases of carcinoma of the breast, operated on in the Middlesex Hospital between 1930 and 1943 and followed up until the end of 1946 (Patey and Dyson, 1948). In 1966 it was thought that it might be of interest to determine the long term results in this same series of cases. The histological records of the Bland Sutton Institute of Pathology were examined, and all cases of carcinoma of the breast operated on during these years from the general wards, either by the writer or by one of his assistants, noted. Excluding cases in which biopsy was the only procedure, records of 146 cases were found. It is not possible at this long interval of time to account for the difference between the present figure of 146 and the figure in the 1948 paper of 118, since the case references of the earlier paper are no longer available. The writer has the strong impression, however, that the earlier paper included only cases operated on by him personally. On the other hand, it became clear when the two series were compared in detail that some cases referred to in the earlier paper did not have a counterpart in the present series. Some may be among records which are unfortunately missing. It is also possible that a few private cases were inadvertently included in the earlier series.

The present paper is therefore a follow up of a slightly different, slightly larger, group of cases, which includes however most of the cases in the 1948 series. In calculating length of survival after operation, only calendar years have been taken into account. Thus, a patient operated on any time in 1930 and dying in 1940 is counted as surviving 10 years. The few patients who died within twelve months of operation have been counted as surviving one year.

### *Selection of Cases and Types of Operation*

During the years when these operations were performed, the general attitude of surgeons towards carcinoma of the breast was that operation was indicated in all cases in which surgery was technically feasible. In addition, there was general agreement that " radical mastectomy ", according to the principles laid down by Halsted and by Handley, was the correct operative procedure. Against this background, there developed in Britain in the 1930's a questioning of the almost automatic performance of " radical mastectomy " for carcinoma of the breast, such questioning however being confined to a small minority. There were three major influences leading to this new attitude. The first and most important was the development and organization of radiotherapy. The second was the growing feeling of dissatisfaction with Sampson Handley's theory of " lymphatic permeation " as the master process in the dissemination of carcinoma of the breast, a theory which in its day provided a logical pathological basis for some of the technical details of " radical mastectomy ". And finally, new techniques for the study of lymphatic anatomy also led to an undermining of some of the postulates on which the operation was based (Gray, 1939).

The writer's main reaction to this movement was the operation of " modified radical mastectomy ". The essential feature of this operation is the removal of the breast and axillary contents in continuity, but without removal of the pectoralis major. The pectoralis minor is however still removed, as in the standard radical operation, as an essential step in complete axillary clearance. Objective demonstration of the efficiency of the axillary clearance by this technique has recently been provided by lymphangiography (Kendall *et al.*, 1963). This operation was performed for the first time on one patient in 1932. It was not adopted, however, as the routine alternative to " standard radical mastectomy " until late in 1936. Thereafter until the end of the period under review, the " standard " radical operation was only performed four times.

A note in the operative record of the 1932 operation provides a small footnote to the introduction of the " modified " radical operation. It reads:

" A slowly growing subareolar tumour in a girl aet 11 which on micro exam proved to be a carcinoma. The breast and pectoral fascia was (sic) removed and the axilla cleared. It was thought that if, as it appeared, the growth was of low malignancy, this would suffice, whereas if of high malignancy the ordinary radical would be of no avail. " (This operation was wrongly referred to in the 1948 paper as a " simple mastectomy ".)

Apart from these two types of " radical " mastectomy, three other types of operation were performed in a small number of cases, but not as routine procedures: simple mastectomy combined with radiotherapy to the axilla; partial mastectomy, usually combined with surgical clearance of the axilla; and radiotherapy of the breast combined with surgical clearance of the axilla. Table I gives the operations performed in the 146 cases.

TABLE I.—*Operations Performed*

| | | |
|---|---|---|
| Standard Radical Mastectomy | 49 | cases |
| Modified Radical Mastectomy | 69 | ,, |
| Simple mastectomy + irradiation of axilla | 17 | ,, |
| Partial mastectomy | 6 | ,, |
| Irradiation of breast + axillary clearance | 5 | ,, |
| Total | 146 | ,, |

Radiotherapy was also given postoperatively in some of the standard and modified radical mastectomy cases, but not on any regular basis of selection except that advanced cases were more likely to be treated. No attempt will be made to take this into account in analysing the results.

### RESULTS

The results in the 146 cases are given in Table II.

If the 4 patients who died of the operation are excluded together with those who died of unknown cause or are untraced, there remain for comparison the 75

TABLE II.—*Results*

| | |
|---|---|
| Alive and well at recent follow up . . | 16 cases |
| Died causes other than carc. of breast . . | 22 ,, |
| Died carc. breast . . . . . . | 75 ,, |
| Died of operation . . . . . . | 4 ,, |
| Untraced and cause of death unknown . | 29 ,, |
| | 146 ,, |

patients who died of the disease, and the 38 patients formed by the first two groups, in which the results might be classified as " good ". Some of the patients, however, who died of other causes did so within a few years of the operation, and hence escaped the risk of late recurrence. On the other hand, some of the patients classified as " untraced " or " cause of death unknown " had survived in good health for many years before they were lost to follow up. We have therefore arbitrarily taken 8 years as a dividing line and excluded from the " good results " 5 patients who died of other diseases earlier than 8 years after operation (at 1, 2, 4, 5, and 7 years), and transferred to the " good results " 5 patients from the " untraced " and " cause of death unknown " groups who survived for 8, 9, 16, 18, and 24 years after operation, and were free of disease when lost to follow up. This adjustment leaves us with the same figures for comparison as in Table II, namely, 75 patients died of the disease and 38 good results.

### *Analysis of Results*

*Died of disease—75 patients*

Thirty-seven patients died of the disease within 2 years of operation, a further 18 within 3 to 5 years, a further 15 within 6 to 10 years, while 5 survived for over 10 years, one patient dying in each of the 11th, 12th, 13th, 16th, and 18th years after operation.

*" Good results "—38 patients* (Table III)

The main features of these 38 cases are summarized in Table III.

TABLE III.—" *Good Results* "

| Case | Year | Histol. No. | Age at op. | Type of op. | Axillary nodes | Result | Cause of death | Comment |
|---|---|---|---|---|---|---|---|---|
| 1 | 1930 | 472 | 43 | Standard Rad. | No note | Died 1945 | Pneumonia | 15 yrs Post. op. Age 58 |
| 2 | 1930 | 843 | 67 | ,, | — | A & W 1939 | Untraced since | 9 yrs ,, Age 76 |
| 3 | 1932 | 336 | 69 | ,, | — | Died 1942 | Pneumonia | 10 yrs ,, Age 79 |
| 4 | 1932 | 908 | 11 | Mod. Rad. | — | A & W 1966 | | 34 yrs ,, Age 45 |
| 5 | 1933 | 53 | 44 | Standard Rad. | — | Died 1955 | Prim. Carc. Ovary | 22 yrs ,, Age 66 |
| 6 | 1933 | 1413 | 40 | ,, | — | A & W 1965 | | 32 yrs ,, Age 72 |
| 7 | 1934 | 350 | 57 | ,, | — | Died 1953 | Carc. Colon | 19 yrs ,, Age 76 |

TABLE III—(*contd*).

| Case | Year | Histol. No. | Age at op. | Type of op. | Axillary nodes | Result | Cause of death | Comment |
|---|---|---|---|---|---|---|---|---|
| 8 | 1934 | 434 | 41 | ,, | — | A & W 1964 | | 30 yrs ,, Age 71 |
| 9 | 1934 | 1151 | 61 | ,, | — | Died 1951 | Myocard. Degen. | 17 yrs ,, Age 78 |
| 10 | 1934 | 1169 | 33 | ,, | + | A & W 1966 | | 32 yrs ,, Age 65 |
| 11 | 1935 | 321 | 57 | ,, | — | Died 1959 | Unknown | 24 yrs ,, Age 81 |
| 12 | 1935 | 1128 | 48 | ,, | + | A & W 1966 | | 31 yrs ,, Age 79 |
| 13 | 1936 | 461 | 53 | ,, | — | Died 1955 | Liver abcess Appendicitis | 19 yrs ,, Age 72 |
| 14 | 1936 | 561 | 46 | ,, | — | Died 1956 | Heart | 20 yrs ,, Age 66 |
| 15 | 1936 | 651 | 46 | Partial Mast. | — | Died 1948 | Glioma brain | 12 yrs ,, Age 58 |
| 16 | 1936 | 1646 | 27 | Standard Rad. | — | A & W 1964 | | 28 yrs ,, Age 55 |
| 17 | 1936 | 1758 | 58 | Mod. Rad. | + | Died 1950 | Carc. Lung | 14 yrs ,, Age 72 |
| 18 | 1937 | 674 | 71 | ,, | — | A & W 1955 | Untraced since | 18 yrs ,, Age 89 |
| 19 | 1937 | 1144 | 52 | Partial Mast. | — | Died 1963 | Carc. Colon | 26 yrs ,, Age 78 |
| 20 | 1939 | 595 | 62 | Mod. Rad. | — | Died 1961 | Heart | 22 yrs ,, Age 84 |
| 21 | 1939 | 1333 | 42 | ,, | + | A & W 1966 | | 27 yrs ,, Age 69 |
| 22 | 1939 | 1782 | 62 | ,, | — | Died 1956 | Heart | 17 yrs ,, Age 79 |
| 23 | 1940 | 127 | 58 | Standard Rad. | — | A & W 1962 | | 22 yrs ,, Age 80 |
| 24 | 1940 | 555 | 56 | Simple mast. and X-rays | + | Died 1964 | Heart | 24 yrs ,, Age 80 |
| 25 | 1941 | 69 | 44 | Mod. Rad. | + | A & W 1964 | | 23 yrs ,, Age 67 |
| 26 | 1941 | 345 | 40 | ,, | + | A & W 1949 | Untraced since | 8 yrs ,, Age 48 |
| 27 | 1941 | 368 | 77 | ,, | — | Died 1952 | Cerebral Haem. | 11 yrs ,, Age 88 |
| 28 | 1941 | 438 | 49 | ,, | + | Died 1949 | Carc. Colon | 8 yrs ,, Age 57 |
| 29 | 1941 | 523 | 32 | ,, | — | A & W 1966 | | 25 yrs ,, Age 57 |
| 30 | 1941 | 850 | 54 | ,, | — | A & W 1966 | | 25 yrs ,, Age 79 |
| 31 | 1942 | 101 | 50 | Simple Mast. and X-rays | No note | A & W 1958 | Untraced since | 16 yrs ,, Age 66 |
| 32 | 1942 | 596 | 51 | Mod. Rad | — | A & W 1964 | | 22 yrs ,, Age 73 |
| 33 | 1942 | 1155 | 47 | ,, | — | A & W 1964 | | 22 yrs ,, Age 69 |
| 34 | 1942 | 1303 | 32 | ,, | — | A & W 1966 | | 24 yrs ,, Age 56 |
| 35 | 1943 | 429 | 56 | ,, | + | A & W 1966 | | 23 yrs ,, Age 79 |
| 36 | 1943 | 495 | 52 | ,, | — | Died 1960 | Ruptured Heart | 17 yrs ,, Age 69 |
| 37 | 1943 | 919 | 44 | ,, | + | A & W 1965 | | 22 yrs ,, Age 66 |
| 38 | 1943 | 1300 | 55 | Simple Mast. and X-rays | No note | Died 1954 | Heart | 11 yrs ,, Age 66 |

+ = Axillary nodes invaded. — = Axillary nodes not invaded. A & W = Alive and well.

In 3 patients the survival time was 8 to 9 years, in 6–10 to 15 years, in 8–16 to 20 years, in 17–21 to 30 years. Four have survived for over 30 years, the longest survival being that of the girl aged 11, who was operated on in 1932. Thirty-four years later (1966) she was well and married with a family.

*Axillary nodes*

Information is available on the axillary nodes in 103 of the 113 cases forming the above two groups, i.e. " died of the disease " and " good results ". In 64 cases the nodes were invaded, and in these the results were good in 10, while 54 patients died of the disease. In 39 cases the nodes were not invaded, and in these the results were good in 25, while 14 patients died of the disease.

These results are in accordance with the general experience that absence of invasion of axillary nodes is a good prognostic factor, but not unfortunately in any absolute sense. At the time when these operations were performed the present practice of searching for and sectioning all axillary lymph nodes had not started, so that further subdivision according to the degree of axillary invasion is not possible. It is probably of significance, however, that, of the 10 good results in spite of axillary node invasion, in 4 cases it was noted that the invasion was " early " and confined to one node (Cases 10, 25, 28, 35. Table III), and in a further case to only two nodes (Case 21).

The improved prognosis associated with absense of invasion of axillary lymph nodes shows itself not only in a lesser liability to die of the disease, but also by death occurring later. Thus, the average survival of the 54 patients with invaded axillary lymph nodes who died of the disease was 3·5 years, while that of 14 patients without invasion of axillary nodes who died of the disease was 7·6 years. The same point is also illustrated in the 5 patients who survived for more than 10 years before dying of the disease. In 4 there was no invasion of axillary lymph nodes, and in the 5th, who died 16 years after operation, " early " invasion of one node only.

*Carcinoma of opposite breast*

In 6 cases of the present series there was bilateral primary carcinoma of the breasts. In 3 cases the patients had had the opposite breast removed for carcinoma previously (2, 7, and 19 years); in 3 cases the carcinoma of the opposite breast developed subsequent to the operations under consideration (2, 3, and 15 years). As regards results, 3 patients died of the disease and one of unknown cause; one was alive and well in 1966, 25 years after the first operation and 10 years after the second; one died of another cause 26 years after the first operation and 7 years after the second.

It is of course not possible to determine which of the two primaries was responsible in those patients who died of the disease. For the purposes of the paper they have been counted as dying from the primary under consideration.

### *Results According to Type of Operation*

In the present series three types of procedure were performed only in selected cases—simple mastectomy, partial mastectomy, and irradiation of the breast with axillary clearance. " Standard " and " modified " radical mastectomies were however performed as routine procedures.

*Standard radical mastectomy*

This operation was performed 49 times. Of the 40 cases available for analysis, 25 patients died of the disease (62·5%), and in 15 (37·5%) the results were good. Information on the axillary nodes is available in 39 of the 40 cases. Out of 22 cases in which the nodes were invaded, in 20 the patients died of the disease and the results were good in 2. Of 17 cases in which the nodes were not invaded, in 5 the patients died of the disease and in 12 the results were good. The result was also good in the one patient about whom no information on the axillary nodes is available. Of the 9 cases not analysed, 2 patients with invaded axillary nodes died of the operation. In 3 of the remaining 7 the nodes were invaded, and in 4 not invaded.

In one of the 2 cases with a good result in spite of invasion of axillary nodes, there is a note that the invasion was confined to the peripheral lymph sinus of one node. This patient was alive and well in 1966, 32 years after operation, aged 65 (Case 10). In the other patient with axillary node invasion who was also alive and well in 1966, 31 years after operation, aged 79, there are no details of the degree of axillary invasion (Case 12).

One case from the good result group is of special interest (Case 8). The patient was operated on in 1934 when she was aged 41, axillary nodes not being invaded. In 1949, 15 years after operation, she developed a pleural effusion shown by biopsy to be carcinomatous, for which she was treated by aspiration and a course of testosterone. In 1955, 21 years after operation, both ovaries were removed and showed deposits of growth compatible with breast origin. In November, 1964, 30 years after operation and aged 71, she was reported as being alive, well, " and working ".

*Modified radical mastectomy*

This operation was performed 69 times. Of the 54 cases available for analysis, in 36 the patients died of the disease (66·7%), and in 18 (33·3%) the results were good. Out of 34 cases in which the axillary nodes were invaded, in 27 the patients died of the disease and the results were good in 7. In 3 of these 7 cases the axillary invasion was confined to one node, and in a further case to two nodes. Of 20 cases in which the axillary nodes were not invaded, 9 patients died of the disease and in 11 the results were good. Of the 15 cases not analysed, 2 patients with invaded axillary nodes died at operation. In 6 of the remaining 13 the nodes were invaded, and in 7 not invaded.

One of the patients in whom the axillary nodes were not invaded (Case 29), following the operation in 1941 at the age of 32, remained well until 1956, when she developed a second primary growth in the opposite breast. This was dealt with by an extended radical operation. At this operation neither the axillary nor the internal mammary nodes were invaded. The patient was alive and well in 1966, aged 57, 25 years after the first operation and 10 years after the second.

*Simple mastectomy+irradiation of the axilla*—17 cases

The main indication for the performance of this operation was the combination of an elderly patient and the absence of clinical evidence of invasion of the axillary lymph nodes. Thus, 8 of the 17 patients were aged 70 or over, 2 of these being

80 or over, while a ninth was aged 69. In addition, in a few patients the operation seems to have been carried out as a palliative procedure.

Of the 9 cases available for analysis, in 6 the patients died of the disease, and in 3 the results were good. One of the patients who died of the disease had been operated on 2 years previously for a carcinoma of the opposite breast with invaded lymph nodes; another had a carcinoma of the opposite breast 2 years after the first operation. This patient died 10 years after the first operation, and 8 years after the second.

Of the 3 cases with good results, one patient was alive and well 16 years after operation (Case 31), and the other 2 died of other causes, one 11 years (Case 38) and the other 24 years (Case 24) after operation, aged 80. In this last case it was noted in the pathological report that the lymph nodes were invaded, but no mention was made of the extent.

Of the 8 cases excluded from analysis, 2 died of other causes in their early eighties within 2 years of operation; a third patient was alive and well at the age of 85, 5 years after operation, when she was lost to follow up.

*Partial mastectomy*—6 cases

In cases in which this operation was performed, the primary growth was small and there was no gross clinical involvement of the axillary lymph nodes. In 5 of the 6 cases an axillary dissection was carried out in addition.

Three patients died of the disease at 1, 2, and 4 years after operation. In 2 of these cases the axillary nodes were invaded, and in the third the axilla was not dissected. Two patients died of other causes at 12 years (Case 15) and 26 years (Case 19) after operation.

The patient excluded from the analysis died of another cause (nephritis) 7 years after operation. She had had the other breast removed for carcinoma 19 years before the operation under consideration. There is one point of special interest in this case, which will be elaborated in the discussion. Two years after the partial mastectomy, the patient had to have the breast removed for recurrence of intra-mammary growth. On pathological examination, three separate nodules of growth were found in the breast.

*Irradiation of the breast and dissection of the axilla*—5 cases

This procedure was carried out for a short time only to explore the potentialities of irradiation as a treatment of the primary growth. All were advanced cases with clinical evidence of invasion of axillary lymph nodes, confirmed on histological examination. All 5 patients died of the disease, 2 at one year, and the other 3 at 3, 7, and 8 years after operation.

### DISCUSSION

The most striking fact emerging from the analysis is that the great majority of the patients died of the disease in spite of treatment. The mortality figure of the present series is of the same order as that of Truscott (1947) who, in an analysis of 1101 cases of carcinoma of the breast operated on at the Middlesex Hospital between 1926 and 1940 inclusive, found that 72% of traced cases had died of the disease. The results in the present series can therefore probably be regarded as typical of those obtaining with the methods of selection and treatment then practiced.

The results of surgical treatment of carcinoma of the breast are now better than they were 20 or 30 years ago, but this is in large part due to a realisation of the limitations of surgery, and a consequent better selection of cases (Haagensen, 1956). However justified and indeed desirable this attitude may be, on a broad view it is merely transferring the problem. In spite of the development of such ancillary forms of therapy as hormonal therapy and surgery, it is still probably true that a woman today developing carcinoma of the breast is much more likely to die of the disease than not.

The prognostic significance of the findings in the axillary nodes, confirmed in the present study, can be expressed in a few generalizations. A woman with advanced invasion of the axillary nodes stands little chance of not dying of the disease, and such death is likely to be sooner rather than later. A woman with absence of invasion of the axillary nodes stands an approximate 60% chance of not dying from the disease, and, should such death occur, it is likely to be later rather than sooner. The fate of a woman with limited axillary invasion confined to one or possibly two nodes may approximate to that of a woman without axillary invasion.

Cases of carcinoma of the breast may thus be divided into two main groups; a group in which axillary invasion is absent or limited, in which the results are relatively good; and a group in which axillary invasion is advanced, in which the results are almost uniformly bad. A theoretical question of great practical importance is whether time or malignancy is the main factor determining the group into which a woman with carcinoma of the breast will fall. If it is time, then the problem is theoretically soluble by earlier diagnosis. If it is malignancy, then appreciable improvement in results depends on the development of new forms of treatment. No certain answer can at present be given to this question. All that can be said from the present study is that the tendency for death from carcinoma to occur several years later in cases in which the axillary nodes are not invaded than in cases in which they are invaded is more easily explicable on the basis of malignancy rather than time being the important factor.

The only two types of operation in the present series in which the results are in any way comparable are standard and modified radical mastectomy, since these were the only operations performed as routine procedures. The good results in standard radical mastectomy—15 out of 40 cases or 37·5%—are marginally better than those of modified radical mastectomy—18 out of 54 cases or 33·3%. However, the standard error of the difference is 4·4%. The observed difference of 4·2% could therefore easily have arisen by chance. The late results of the two operations on the present evidence are therefore of the same order. If any difference exists, it will require a larger and properly controlled series of cases for its demonstration.

The above comparison is based only on mortality from the disease. If further investigation should confirm that the results on this basis of the two operations are of the same order, the morbidity, as illustrated by such features as local recurrence, which still remains disturbingly high (Truscott, Philip, 1967), or physical and psychological upset, might tilt the balance in favour of one or other operation.

No conclusions can be drawn from the present study on the position of simple mastectomy combined with irradiation of the axilla in the treatment of carcinoma of the breast. One comment may however be made. Under the term " simple mastectomy " are included in practice two distinct procedures—removal of the main part of the breast only, and removal of the breast plus the axillary tail. The latter procedure involves as an almost inevitable concomitant removal of the pectoral group of lymph nodes. Thus, Brinkley and Haybittle (1966), in reporting the interim results of a recent controlled clinical trial at Cambridge, noted that in all but 24 of 113 cases of " simple " mastectomy axillary lymph nodes were available for histology. A " simple mastectomy " of this type should of itself deal satisfactorily with the pathology in the favourable type of case, i.e. the case in which the axillary nodes are either not invaded, or in which the invasion is limited to one or two nodes. The part that irradiation plays in the combined procedure would therefore seem to warrant further critical investigation.

As regards partial mastectomy, mention was made of one case in the present series in which removal of the breast was subsequently performed because of recurrence of growth in the breast. In the 1948 paper, another similar case was also mentioned. As a result of this experience and of " other similar cases in a private series ", it was then concluded that " while in an occasional case partial mastectomy combined with a dissection of the axilla might be justifiable . . . the danger of further development of carcinoma in the remaining breast tissue rendered the procedure an unwise routine ". Since partial mastectomy has recently again found an advocate (Porritt, 1964), it may be appropriate to add here a summary of the main facts of the private series referred to above.

Between 1930 and 1943 the writer performed the operation of partial mastectomy in private in all 5 times. In 4 cases the axillary nodes were not invaded, in one the axilla was not dissected. In addition, in 1958 he operated on a patient who 15 years before had had a partial mastectomy without axillary dissection performed by another surgeon. The patient had developed a further obvious carcinoma of the breast, which was dealt with by modified radical mastectomy. The axillary nodes were not invaded.

Including this patient, in 3 of the 6 private patients removal of the breast because of the further development of carcinoma in it was carried out at 6, 14, and 15 years after the partial mastectomy. In all 3 cases, death from carcinoma eventually took place at 1 year, 14, and 5 years respectively after the second operations. In the 2 cases in which the writer performed the original partial mastectomy, the pathologist was able to compare the histology of the growths removed at the earlier and later operations, and expressed the opinion that they represented independent primary growths. In light of all these facts the writer feels confirmed in his opinion that partial mastectomy is justified in carcinoma of the breast, if at all, only in exceptional circumstances.

There remains for brief comment the question of second primary carcinoma in the opposite breast. The true risk of this happening is difficult to express owing to the progressively diminishing population at risk resulting from deaths from carcinoma and other causes. In the present material it is impossible, since in 3 of the 6 examples the carcinoma of the opposite breast preceded the operation under consideration. One can say, however, that, compared with the risk that a woman runs from the carcinoma she has already developed, the risk she runs from a carcinoma which might develop in the opposite breast is minor. Were a substantial improvement in the results of treatment of carcinoma of the breast to take place, then the question would assume a greater importance. But in this case the results of the improvement in treatment would also apply to the carcinoma of the second breast.

To conclude, the main impression left after this follow up of cases of carcinoma of the breast must be one of dissatisfaction. The important fact is that the great majority of the patients died of the disease in spite of treatment. The approximately similar proportion of good results in the two main types of operative procedure suggests that both are skimming off a common favourable group. The hypothesis that the size of the problem could be appreciably reduced by early diagnosis still lacks firm foundation, but for the present must continue to be a basis for action. The solution of the main problem, however, in the breast as in other parts of the body, probably depends on a deepening of our understanding of the malignant process and its possible controls.

### SUMMARY

1. The results in 146 cases of carcinoma of the breast operated on in the years 1930 to 1943 inclusive by the writer or his assistants have been reviewed.

2. Excluding patients untraced, those in whom the cause of death was unknown, who died of causes other than carcinoma of the breast within 8 years, or who died of operation, 113 cases remain for analysis.

3. Of these 113 cases, 75 patients died of the disease, and in 38 the result was "good".

4. The moderately good prognosis of absence of invasion of the axillary lymph nodes is confirmed. In addition, if a patient without invasion of the axillary nodes does die of the disease, death tends to be appreciably later than in patients with axillary node invasion.

5. Bilateral carcinoma of the breast occurred in 6 patients.

6. "Good" results were obtained in 15 out of 40 cases in which standard radical mastectomy was performed (37·5%), and in 18 out of 54 cases in which modified radical mastectomy was performed (33·3%). The difference is not statistically significant.

7. Subsequent removal of the breast because of further manifestations of carcinoma was necessary in 1 of the 6 cases of partial mastectomy of the present series. It was also necessary for the same reason in 3 out of 6 cases of partial mastectomy in a personal private series.

I wish to acknowledge my indebtedness to Mr. Cowan, Records Officer to the Middlesex Hospital and to Miss Curtis his Deputy; also to Miss Andrea Donagh and Miss Gillian Davies, Medical Students, for their patient searches in the histological records.

### REFERENCES

BRINKLEY, D. AND HAYBITTLE, J. L.—(1966) *Lancet*, ii, 291.
GRAY, J. H.—(1939) *Br. J. Surg.*, **26**, 462.
HAAGENSEN, C. D.—(1956) 'Diseases of the Breast'. Philadelphia (Saunders), p. 625.
KENDALL, B. E., ARTHUR, J. F. AND PATEY, D. H.—(1963) *Cancer, N.Y.*, **16**, 1233.
PATEY, D. H. AND DYSON, W. H.—(1948) *Br. J. Cancer*, **2**, 7.
PHILIP, J. F.—(1967) *Br. med. J.*, i, 323.
PORRITT, A.—(1964) *Br. J. Surg.*, **51**, 214.
TRUSCOTT, B. McN. (1947) *Br. J. Cancer*, **1**, 129.